# Comparative Effects of Pairwise Combinations of Carboplatin, Everolimus, and Astaxanthin on Cell Viability, Migration, and Inflammatory Biomarkers in SKOV3 Ovarian Cancer Cells

**DOI:** 10.3390/ijms27187995

**Published:** 2026-09-08

**Authors:** Mete Hakan Karalok, Burcu Biltekin, Ebru Karci, Hafize Uzun

**Affiliations:** 1 Department of Obstetrics and Gynecology, Faculty of Medicine, Atlas University, Istanbul 34408, Türkiye; 2 Department of Histology and Embryology, Faculty of Medicine, Atlas University, Istanbul 34408, Türkiye; burcu.biltekin@atlas.edu.tr; 3 Department of Oncology, Faculty of Medicine, Atlas University, Istanbul 34408, Türkiye; dr.ebrkarc@yahoo.com.tr; 4 Department of Biochemistry, Faculty of Medicine, Atlas University, Istanbul 34408, Türkiye; hafize.uzun@atlas.edu.tr

**Keywords:** ovarian cancer, SKOV3, everolimus, carboplatin, astaxanthin, cell viability, cell migration, defensins, TNF-α, IL-1β

## Abstract

Ovarian cancer remains a major cause of gynecological cancer-related mortality, highlighting the need for novel therapeutic strategies capable of enhancing antitumor activity while targeting multiple mechanisms involved in tumor progression. Everolimus, an inhibitor of the mammalian target of rapamycin pathway, and carboplatin are established antineoplastic agents with distinct mechanisms of action, whereas astaxanthin is a bioactive carotenoid with antioxidant, anti-inflammatory, and potential anticancer properties. This study compared the individual and pairwise combination effects of carboplatin, everolimus, and astaxanthin on cell viability, migratory capacity, and inflammatory and tumor-associated biomarkers in SKOV3 ovarian cancer cells. Human SKOV3 ovarian cancer cells were cultured under standard conditions and exposed to different concentrations of carboplatin, everolimus, and astaxanthin, either individually or as pairwise two-drug combinations, for 24–72 h. Cell viability was assessed using the Cell Counting Kit-8 (CCK-8) assay. Cell migration was evaluated using a scratch wound-healing assay. Based on the experimental treatment protocol, 100 nM everolimus, 100 μM carboplatin, and 100 μM astaxanthin, alone and in combination, were further evaluated for their effects on β-defensin 1, β-defensin 2, α-defensin 1, tumor necrosis factor-alpha (TNF-α), and interleukin-1 beta (IL-1β) levels. Biomarker concentrations were determined by ELISA. Experiments were independently performed in triplicate. Everolimus, carboplatin, and astaxanthin produced concentration- and time-dependent alterations in SKOV3 cell viability, with more pronounced cytotoxic effects generally observed following prolonged exposure. The pairwise combination treatments also substantially affected cell viability, particularly at later experimental time points. Assessment of migratory capacity demonstrated significant differences in wound closure among treatment groups at 24, 48, and 72 h (*p* = 0.0224, *p* = 0.0155, and *p* = 0.0028, respectively), indicating a time-dependent inhibitory effect on SKOV3 cell migration. Treatment with carboplatin, everolimus, and astaxanthin, individually or in pairwise combinations, also significantly modulated β-defensin 1, β-defensin 2, α-defensin 1, TNF-α, and IL-1β levels compared with control cells, with the magnitude and direction of these changes varying according to treatment regimen and exposure duration. Everolimus, carboplatin, and astaxanthin exerted significant time- and treatment-dependent effects on the viability and migratory behavior of SKOV3 ovarian cancer cells and markedly modulated defensin and pro-inflammatory cytokine responses. The findings demonstrate distinct treatment- and time-dependent responses to the individual agents and their pairwise combinations in SKOV3 ovarian cancer cells. These observations represent preliminary comparative in vitro effects rather than evidence of pharmacological synergy or therapeutic benefit. Further mechanistic studies using additional ovarian cancer cell lines, formal drug interaction analyses, and in vivo models are required to establish the biological and potential therapeutic relevance of these treatment combinations.

## 1. Introduction

Ovarian cancer remains one of the most challenging gynecological malignancies because of its frequent presentation at an advanced stage, high recurrence rate, and substantial mortality. Despite advances in molecularly targeted therapies and maintenance strategies, cytoreductive surgery followed by platinum-based chemotherapy remains a cornerstone of treatment for advanced ovarian cancer. However, the development of intrinsic or acquired platinum resistance represents a major therapeutic obstacle and is strongly associated with disease recurrence and poor clinical outcomes [1,2,3]. Consequently, therapeutic approaches that enhance the antitumor activity of platinum compounds or simultaneously target complementary pathways involved in tumor cell survival and progression remain of considerable interest.

Carboplatin is one of the principal platinum compounds used in ovarian cancer treatment. Its antitumor activity is primarily mediated through platinum–DNA adduct formation, resulting in DNA damage and ultimately inhibition of tumor cell proliferation. Nevertheless, ovarian cancer cells can acquire resistance through multiple mechanisms, including alterations in DNA damage repair, apoptosis, intracellular drug accumulation, cellular stress responses, and prosurvival signaling pathways [2,4]. Experimental studies using SKOV3 cells have also demonstrated the development of carboplatin-resistant phenotypes, supporting the usefulness of this cell line for investigating mechanisms and potential strategies related to platinum resistance [4].

The phosphatidylinositol-3-kinase (PI3K)/AKT/mechanistic target of the rapamycin (mTOR) signaling pathway is particularly relevant in this context because it regulates several processes essential for malignant progression, including cell growth, proliferation, survival, metabolism, and treatment response. Everolimus (RAD001), a pharmacological inhibitor of mTOR, has therefore attracted attention as a potential component of combination anticancer strategies. Previous experimental evidence demonstrated that everolimus inhibited proliferation of ovarian cancer cells with activated AKT/mTOR signaling, including SKOV3 cells, and enhanced platinum-induced apoptosis. In SKOV3 xenograft models, mTOR inhibition also reduced tumor growth, angiogenesis, intraperitoneal dissemination, and ascites formation while enhancing the therapeutic activity of platinum treatment [5]. These findings provide a mechanistic rationale for evaluating everolimus together with platinum-based agents in ovarian cancer.

Astaxanthin is a naturally occurring xanthophyll carotenoid characterized by antioxidant, anti-inflammatory, and potential antineoplastic properties. Experimental studies suggest that its anticancer effects involve multiple molecular mechanisms, including modulation of oxidative stress, apoptosis, cell cycle regulation, and signaling pathways such as NF-κB and MAPK [6]. Of particular relevance, astaxanthin has previously demonstrated antiproliferative and antimigratory effects in SKOV3 ovarian cancer cells. Su et al. reported that astaxanthin-based treatment inhibited proliferation and migration, affected apoptosis-related signaling, and influenced drug-resistant characteristics in SKOV3 cells [7]. These observations raise the possibility that astaxanthin could modify the cellular response to conventional chemotherapy and targeted mTOR inhibition.

In addition to tumor-intrinsic survival pathways, inflammatory and innate immune mediators are increasingly recognized as components of ovarian cancer biology. TNF-α and IL-1β can participate in complex tumor–microenvironment interactions and have been shown to influence ovarian tumor cell growth and inflammatory signaling [8,9]. Defensins, including α-defensins (HNP1–4, mainly from neutrophils) and β-defensins (HBD1–4, mainly from epithelial cells), are small host-defense peptides traditionally recognized for their antimicrobial functions but increasingly implicated in cancer-related processes such as cell proliferation, apoptosis, immune-cell recruitment, angiogenesis, and tumor progression. Importantly, the biological effects of defensins appear to be context dependent and may differ according to tumor type and the specific defensin involved [10,11,12]. Recent pan-cancer evidence has further demonstrated cancer-specific alterations in DEFB1 expression and associations with tumor biology and clinical outcomes, supporting the potential relevance of defensin signaling in malignant disease [13].

Based on these observations, the present study was designed to comparatively evaluate three mechanistically distinct agents—carboplatin, everolimus, and astaxanthin—administered individually and in pairwise two-drug combinations. Carboplatin was included as a clinically established platinum-based agent, everolimus as an mTOR-targeted agent with a mechanistic rationale for modulating platinum responsiveness, and astaxanthin as an experimental bioactive compound with reported antiproliferative, antimigratory, and anti-inflammatory properties. Accordingly, we evaluated the pairwise combinations of carboplatin–everolimus, carboplatin–astaxanthin, and everolimus–astaxanthin. The study aimed to compare their effects on SKOV3 cell viability and migratory capacity and to characterize treatment-associated changes in the inflammatory and tumor-associated mediators α-defensin 1 (DEFA1), β-defensin 1 (DEFB1), β-defensin 2 (DEFB2), TNF-α, and IL-1β. This experimental design was intended to explore and compare pairwise biological treatment effects rather than to evaluate a three-drug regimen or establish pharmacological synergy.

## 2. Results

### 2.1. Effects of Individual and Pairwise Treatments with Carboplatin, Everolimus, and Astaxanthin on SKOV3 Cell Viability

**Figure 1:**
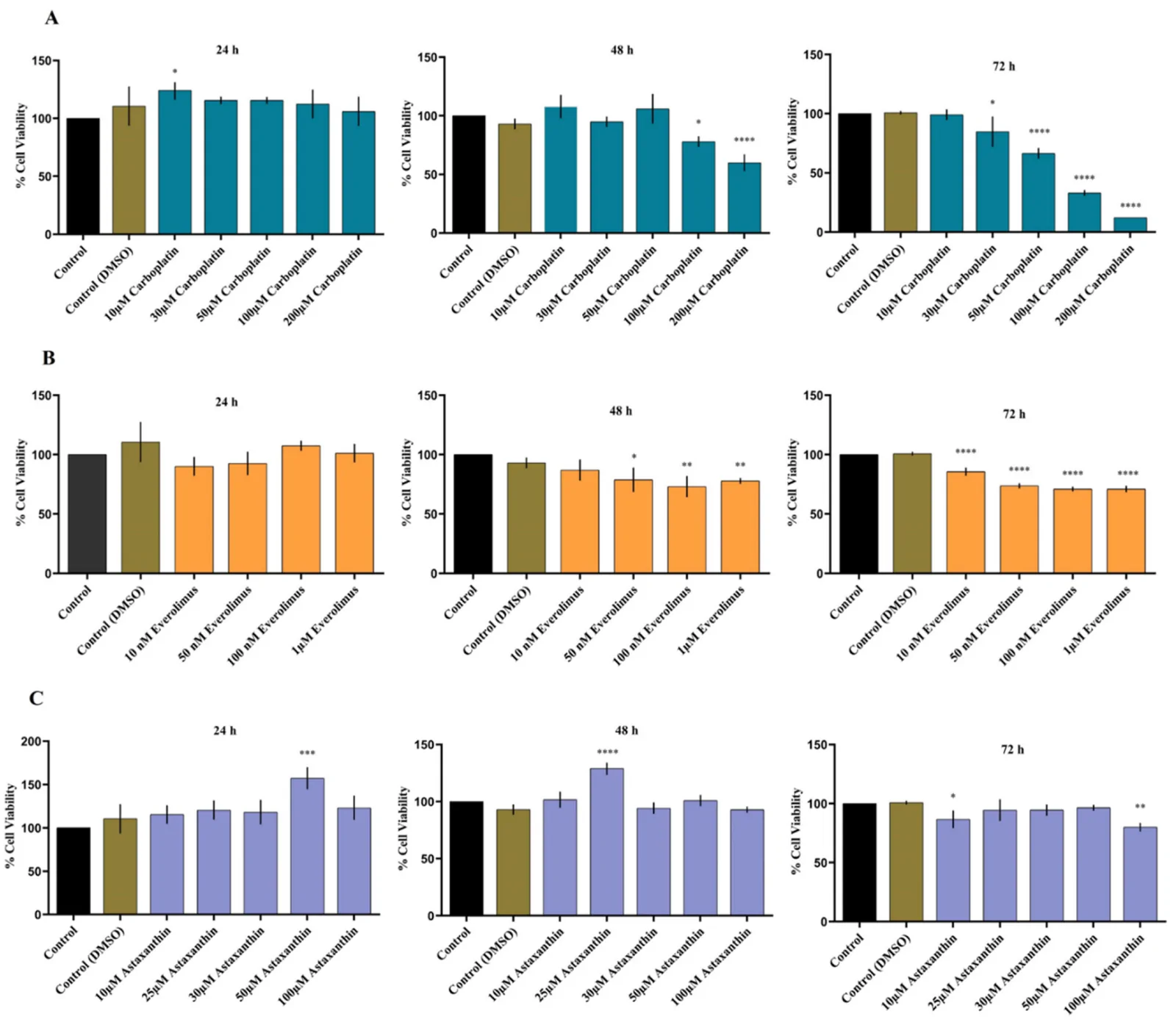
Concentration- and time-dependent viability profiles of SKOV3 cells following individual treatment with carboplatin (**A**), everolimus (**B**) or astaxanthin (**C**). This figure illustrates the cytotoxic effects of everolimus, carboplatin and astaxanthin on the SKOV3 ovarian cancer cell line. Cell viability was monitored over a 24–72 h period using the CCK-8 assay. Dose–response curves for each agent administered as monotherapy, demonstrating a concentration-dependent reduction in cell viability. (* *p* < 0.05, ** *p* < 0.01, *** *p* < 0.001, **** *p* < 0.0001 vs. control).

Concentration- and time-dependent viability profiles of SKOV3 cells presented in Figure 1.

**Figure 2:**
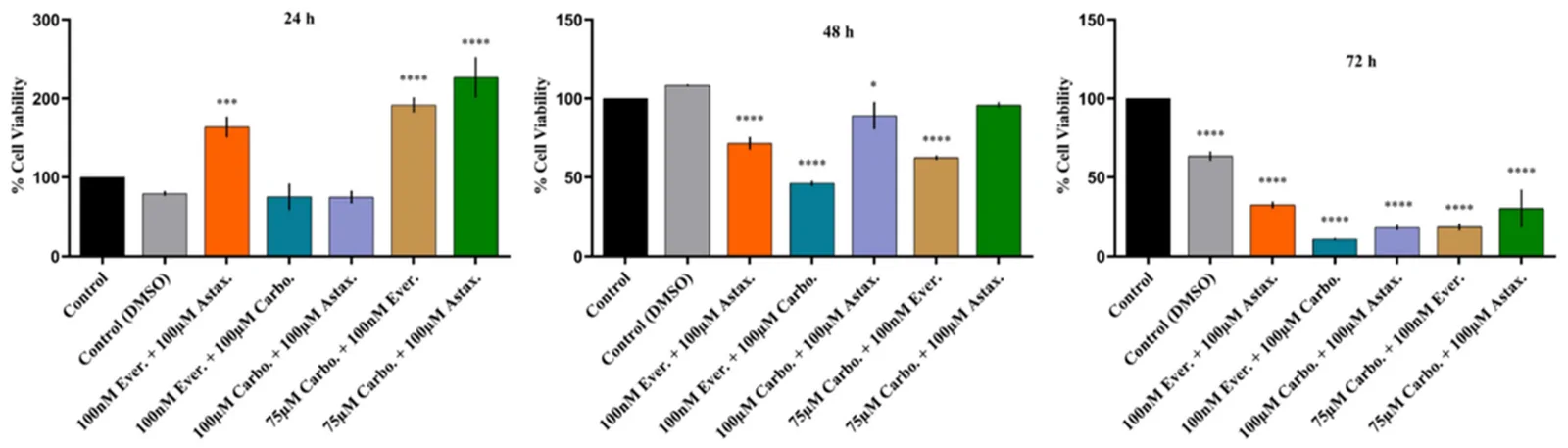
Time-dependent effects of pairwise two-drug combinations of carboplatin, everolimus, and astaxanthin on SKOV3 cell viability. SKOV3 cells were treated with carboplatin–everolimus, carboplatin–astaxanthin, or everolimus–astaxanthin combinations, and cell viability was assessed by the CCK-8 assay after 24, 48, and 72 h. Cell viability is expressed as the percentage of viable cells relative to untreated controls. Data are presented as mean ± SD. (* *p* < 0.05, *** *p* < 0.001, **** *p* < 0.0001 vs. control).

Time-dependent effects of pairwise two-drug combinations of carboplatin, everolimus, and astaxanthin on SKOV3 cell viability presented in Figure 2.

### 2.2. Inhibitory Effects on SKOV3 Cell Migration

**Figure 3:**
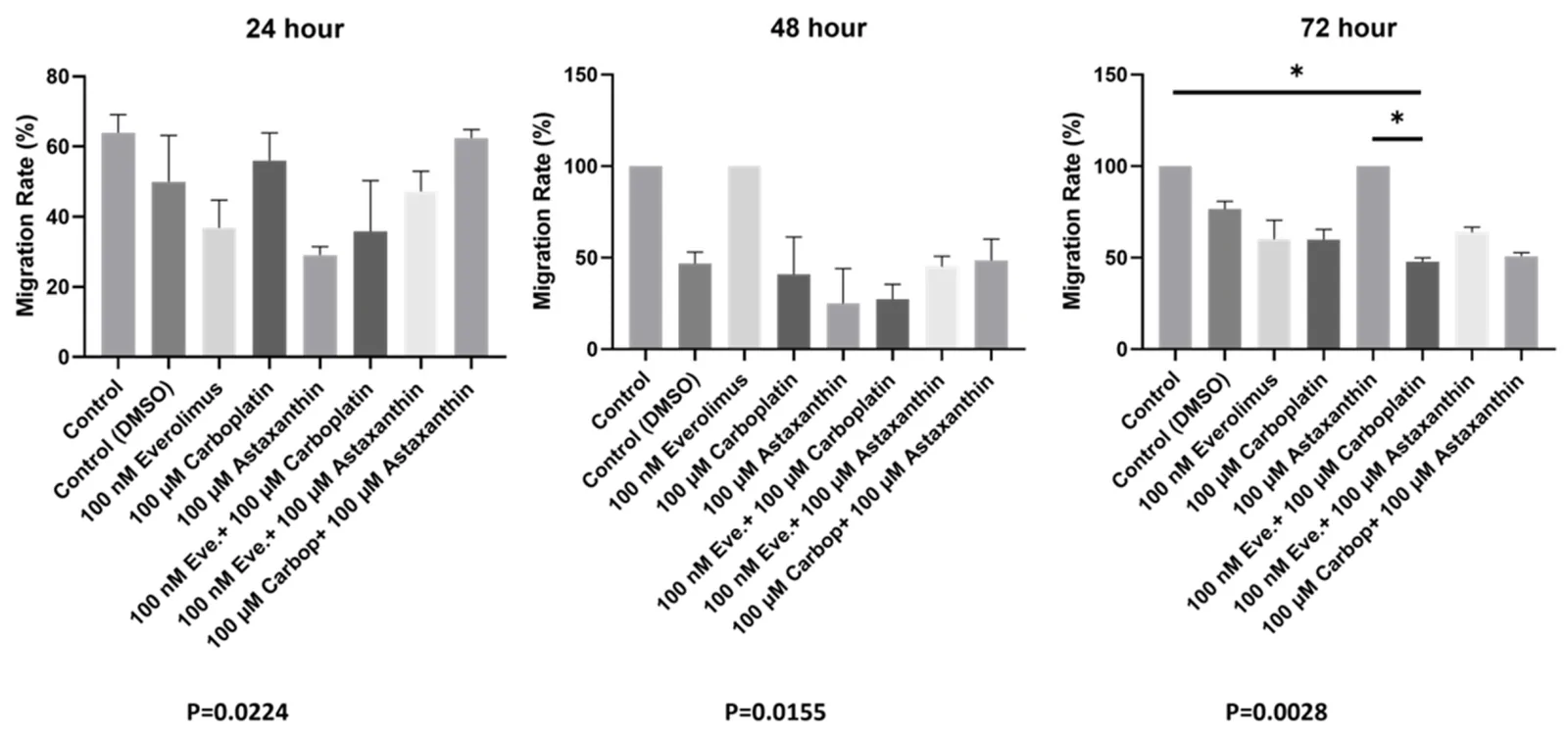
Effects of different treatment doses on cell migration determined by the scratch wound healing assay at 24, 48, and 72 h. Cell migration is expressed as the percentage of wound closure (% migration). Bars represent the mean ± SD of independent experiments. Migration rates varied among the treatment groups over time, with the most pronounced reduction observed at 48 h, followed by partial recovery at 72 h. (* *p* < 0.05, vs. control).

Quantitative analysis of wound closure (Figure 3) demonstrated significant differences in SKOV3 cell migration among cells exposed to carboplatin, everolimus, and astaxanthin individually or in pairwise combinations compared with the untreated control. A statistically significant reduction in wound closure was observed at 24 h (*p* = 0.0224), 48 h (*p* = 0.0155), and 72 h (*p* = 0.0028). The increasing level of statistical significance over time suggests that the inhibitory effect of the treatments on cell migration became more pronounced during the experimental period.

**Figure 4:**
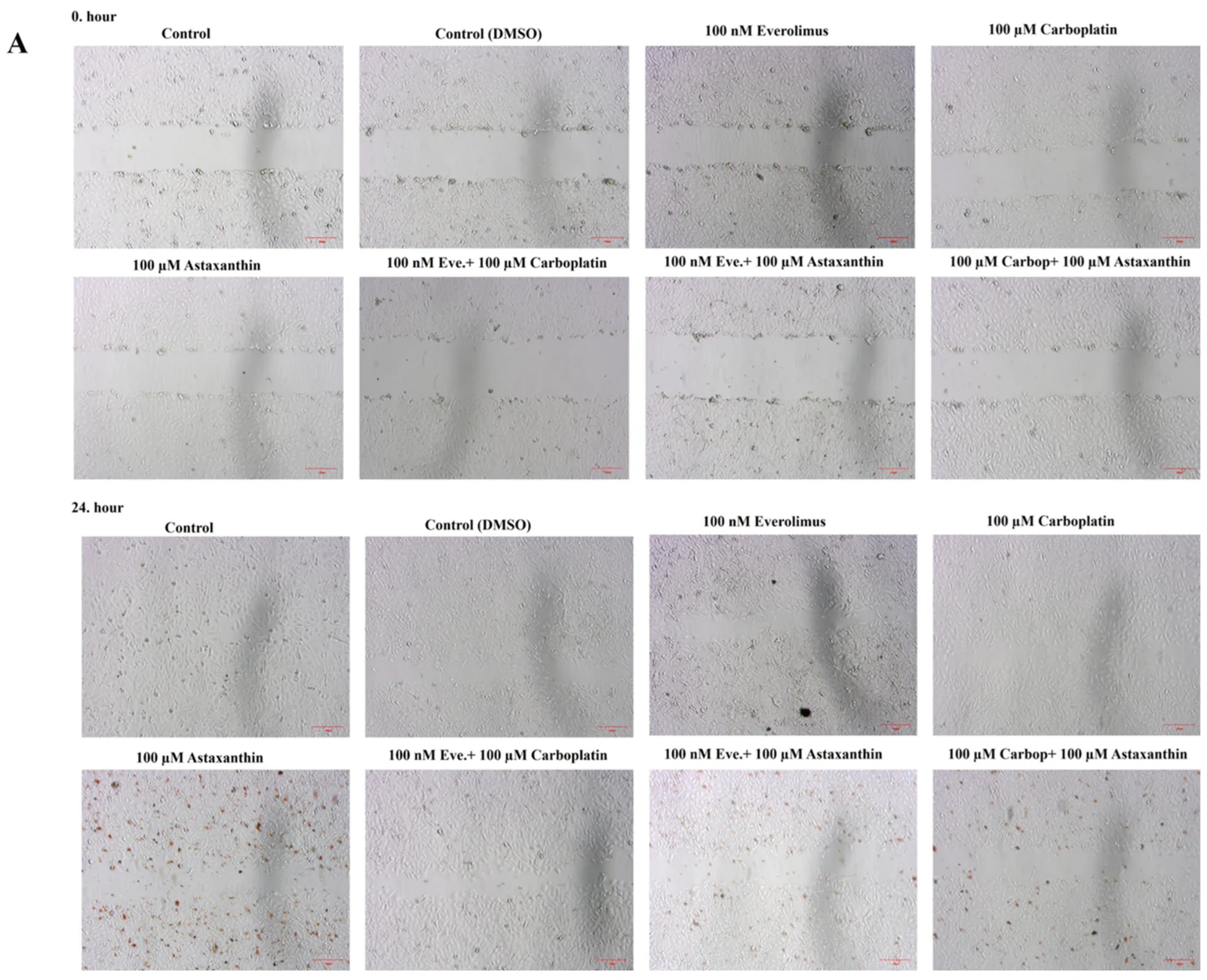

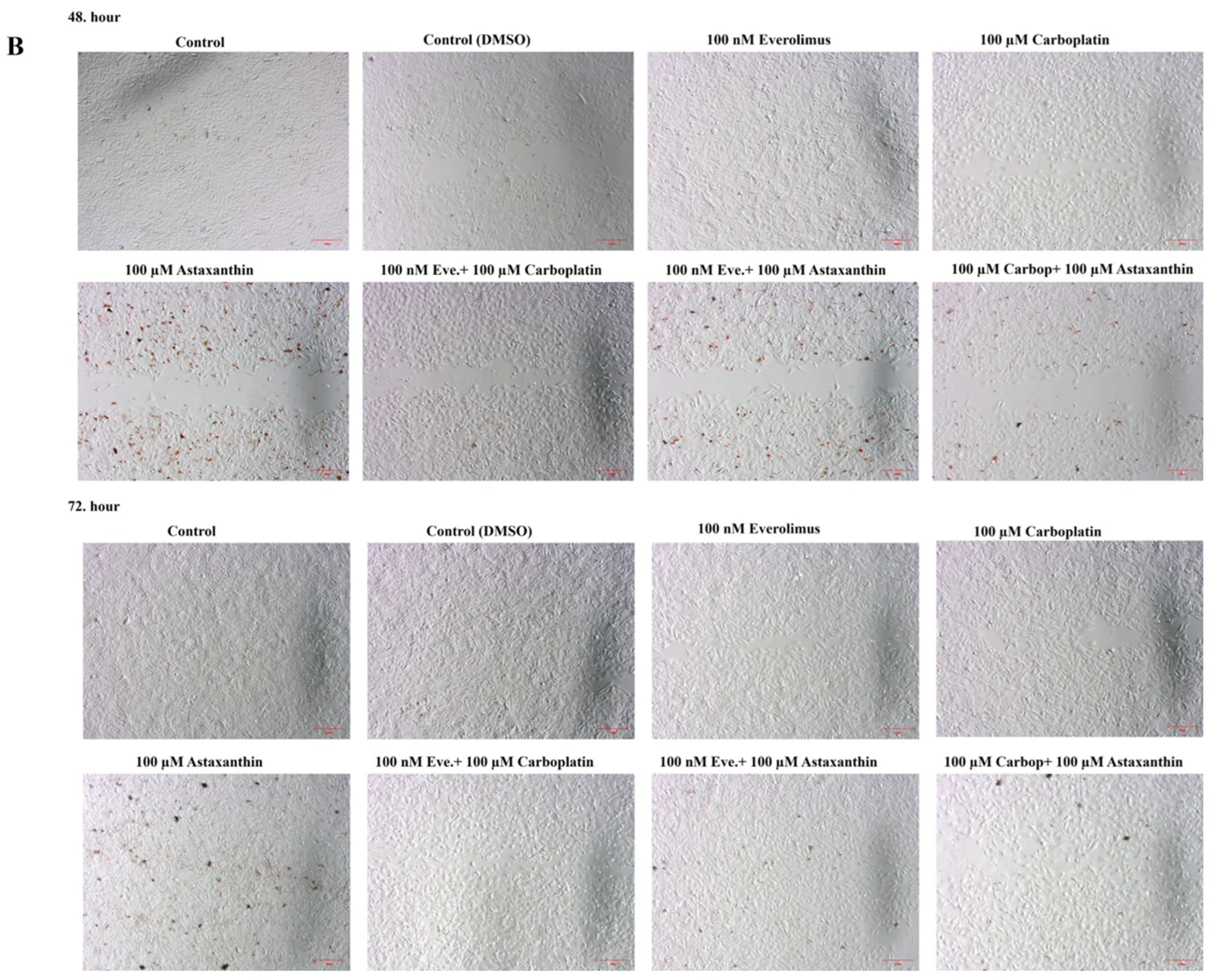
(**A**,**B**) Representative wound-healing images of SKOV3 cells at 0, 24, 48 and 72 h. Scale bars represent 100 µm.

As shown in Figure 4A,B, the wound healing assay revealed differences in the migratory capacity of SKOV3 cells among the experimental groups. Representative images acquired at ×40 magnification demonstrated the progressive closure of the scratch area following treatment. Compared with the control group, treatment with the indicated agents resulted in a noticeable alteration in wound closure, suggesting a modulation of SKOV3 cell migration. Overall, these findings indicate that the treatment may affect the migratory potential of SKOV3 cells.

### 2.3. Modulation of Inflammatory and Tumor-Associated Biomarkers

**Figure 5:**
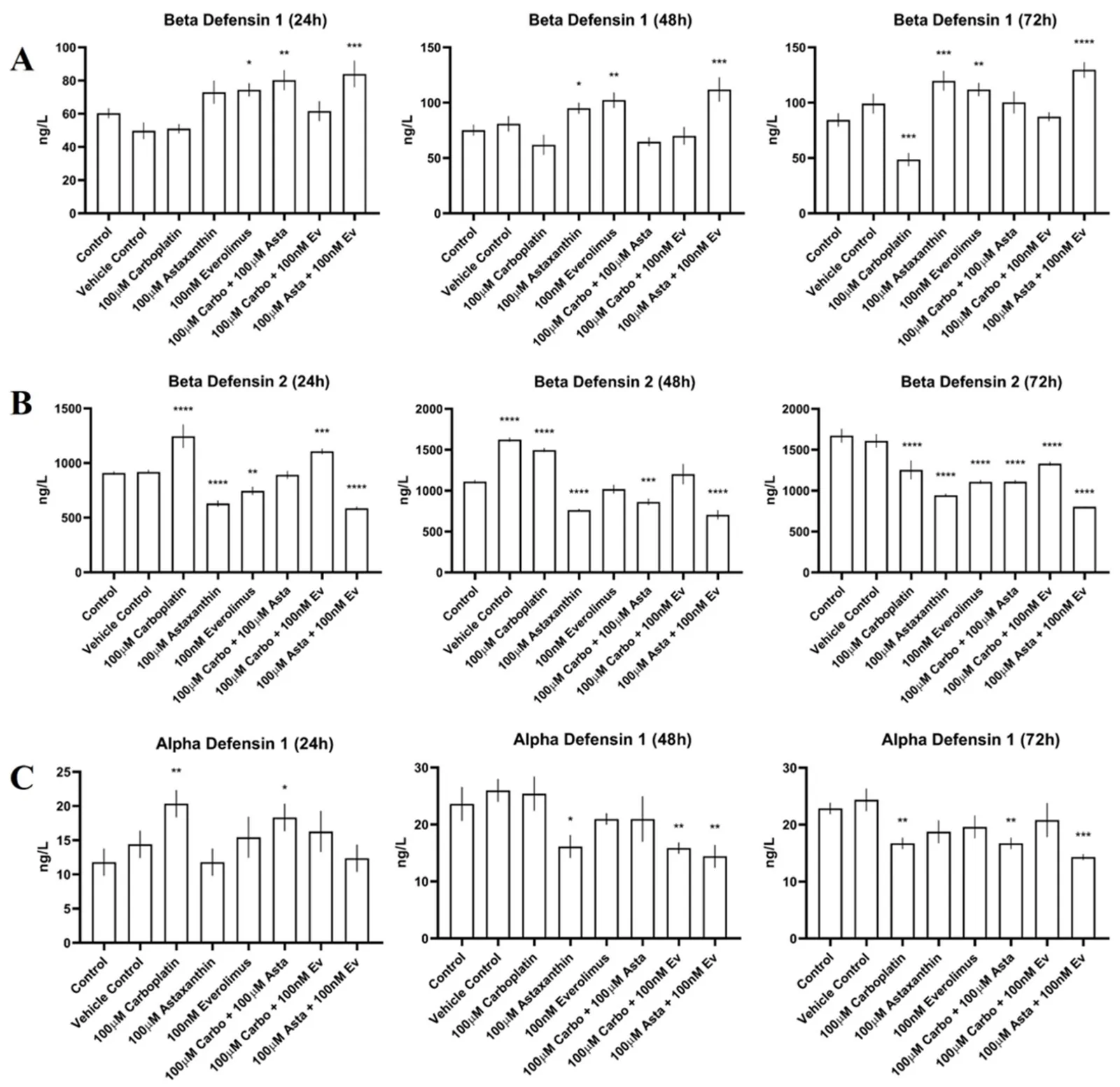
(**A**–**C**) Effects of 100 μM carboplatin (Carbo), 100 μM astaxanthin (Asta), 100 nM everolimus (EV), and their combinations on β-defensin 1 (**A**), β-defensin 2 (**B**), and α-defensin 1 (**C**) levels after 24, 48, and 72 h of treatment. Defensin concentrations were determined by ELISA and expressed as ng/L. Data are presented as mean ± SD. * *p* < 0.05, ** *p* < 0.01, *** *p* < 0.001, **** *p* < 0.0001 vs. the control group.

**Figure 6:**
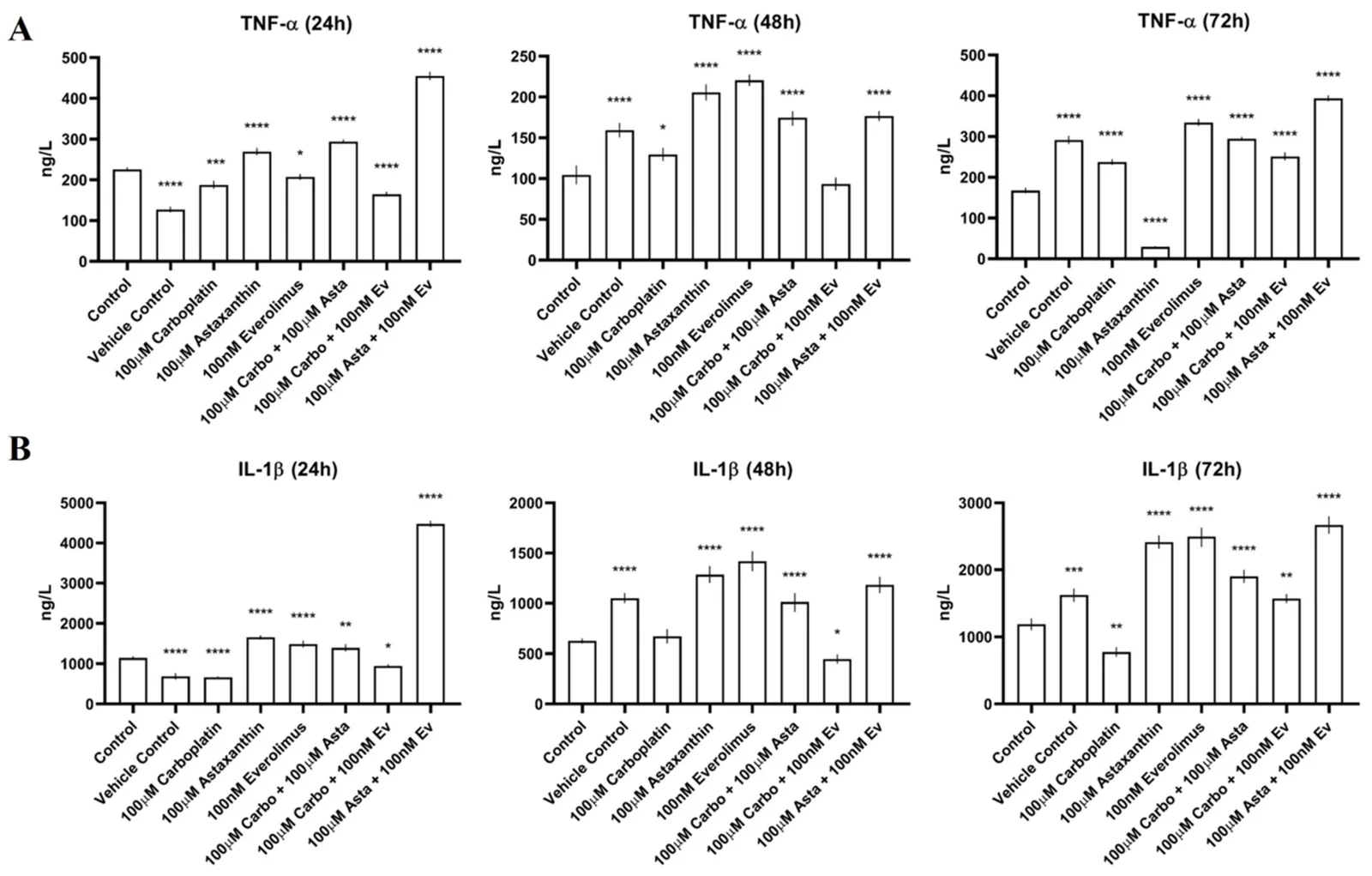
(**A**,**B**). Effects of 100 μM carboplatin (Carbo), 100 μM astaxanthin (Asta), 100 nM everolimus (EV), and their combinations on TNF-α (**A**) and IL-1β (**B**) levels following 24, 48, and 72 h of treatment. Cytokine concentrations were determined by ELISA and expressed as ng/L. Data are presented as mean ± SD. * *p* < 0.05, ** *p* < 0.01, *** *p* < 0.001, **** *p* < 0.0001 vs. the control group.

The effects of carboplatin, everolimus, and astaxanthin, administered individually or in pairwise combinations, on β-defensin 1, β-defensin 2, α-defensin 1, TNF-α, and IL-1β protein levels were evaluated in SKOV3 cell lysates. As shown in Figure 5 and Figure 6, the individual treatments and pairwise combinations significantly altered the levels of these mediators compared with control cells, with treatment- and time-dependent differences in the magnitude and direction of the responses.

## 3. Discussion

The principal finding of the present study was that carboplatin, everolimus, and astaxanthin, administered individually or in pairwise combinations, produced distinct treatment- and time-dependent changes in CCK-8 activity, wound closure, and inflammatory and tumor-associated biomarker levels in SKOV3 ovarian cancer cells. The responses were not uniform across treatments or exposure periods, and the pairwise combinations did not consistently produce greater effects than the corresponding individual agents. These observations indicate that the biological responses to the tested compounds depend on both treatment composition and duration of exposure. Importantly, because formal drug interaction analyses were not performed, the observed differences among pairwise combinations should be interpreted as comparative treatment effects rather than evidence of pharmacological synergy.

Closer examination of the viability profiles revealed a complex temporal response. At 24 h, several carboplatin-containing treatment conditions produced CCK-8 signals above those of untreated cells, whereas other treatment conditions, including some everolimus–carboplatin combinations, already showed reduced CCK-8 activity. By 48 h, the signal had declined in several carboplatin-containing groups, and by 72 h, substantial reductions were apparent across the majority of individual and pairwise treatment conditions. Everolimus and astaxanthin likewise showed relatively preserved or increased CCK-8 activity at earlier time points in some conditions, followed by considerably lower activity after prolonged exposure. Thus, describing these findings simply as progressively increasing cytotoxicity would not adequately reflect the observed treatment-specific patterns.

The early increases in CCK-8 signal, including values exceeding 150% of the untreated control in some conditions, should not be interpreted unequivocally as stimulation of cell proliferation. The CCK-8 assay depends on cellular dehydrogenase activity and therefore primarily reflects metabolic activity associated with viable cells rather than directly measuring DNA synthesis or cell division. Consequently, values above the untreated control could reflect transient increases in metabolic activity, changes in viable cell number, or an adaptive cellular response to treatment. The subsequent transition toward markedly reduced CCK-8 activity at 72 h is compatible with a delayed treatment-associated cytotoxic response following an early adaptive phase. However, the present experiments cannot establish the mechanism underlying this apparent biphasic pattern. Direct assessment of proliferation, apoptosis, cell cycle distribution, and cellular metabolism would be required to distinguish among these possibilities.

The reduction in SKOV3 cell viability observed following carboplatin treatment is consistent with its established cytotoxic activity in ovarian cancer. However, the effectiveness of platinum compounds is frequently limited by the development of drug resistance involving multiple prosurvival mechanisms, including activation of the PI3K/AKT/mTOR pathway. Previous experimental studies have shown that inhibition of PI3K signaling can increase the sensitivity of ovarian cancer cells to carboplatin, with combined PI3K inhibition and carboplatin producing greater suppression of tumor cell survival than either treatment alone [14]. Supporting the potential value of combination strategies, Hasan et al. [15] demonstrated that pretreatment with the natural bioactive compound quercetin enhanced cisplatin cytotoxicity in cisplatin-resistant SKOV3 cells. This effect was associated with increased oxidative stress, activation of mitochondrial apoptosis, and suppression of mTOR/STAT3 signaling. Together, these findings indicate that targeting survival and stress-response pathways may improve the responsiveness of ovarian cancer cells to platinum compounds. In this context, the present findings provide a biological rationale for further evaluation of distinct pairwise approaches in which platinum chemotherapy is combined with either mTOR-targeted treatment or a bioactive natural compound.

Similarly, mTOR signaling has been closely associated with platinum resistance, providing a mechanistic rationale for targeting this pathway in combination with platinum-based chemotherapy. In platinum-resistant ovarian cancer cells, inhibition of mTOR signaling has been shown to enhance responsiveness to platinum agents and reduce tumor cell survival [16]. Supporting this concept, David-West et al. [17] demonstrated that platinum-resistant ovarian cancer cells exhibit mTOR-dependent selective translation of mRNAs involved in DNA damage responses and cell survival. Importantly, simultaneous inhibition of mTORC1 and mTORC2 disrupted the translation of these prosurvival transcripts and re-sensitized resistant ovarian cancer cells to platinum treatment. These findings provide a mechanistic basis for overcoming platinum resistance through mTOR pathway inhibition. Nevertheless, although carboplatin–everolimus produced marked responses under some experimental conditions in the present study, its effects were not consistently greater than those of the corresponding single agents across all time points. Therefore, our findings support further investigation of this combination but do not demonstrate synergistic or superior activity.

The effects of astaxanthin observed in our experiments are also supported by previous evidence demonstrating its anticancer activity in ovarian cancer cells. Su et al. [7] investigated astaxanthin in SKOV3 cells and showed that astaxanthin-based treatment suppressed cell proliferation and migration, promoted cell cycle arrest and apoptosis, and affected signaling pathways including NF-κB and MAPKs. Importantly, their findings also suggested an effect on drug-resistant characteristics of SKOV3 cells [7]. In the present study, astaxanthin alone and in separate pairwise combinations with carboplatin or everolimus affected cell viability and wound-healing responses over time. However, the astaxanthin-containing combinations did not uniformly enhance the effects of the conventional or targeted agents. Rather, their responses varied according to both the treatment partner and exposure duration. The carboplatin–astaxanthin combination may therefore be considered an exploratory model for evaluating astaxanthin alongside platinum exposure, whereas everolimus–astaxanthin represents a separate non-platinum experimental strategy. These findings should not be interpreted as demonstrating that astaxanthin potentiates either carboplatin or everolimus in the absence of formal drug interaction analyses.

The inhibition of cell migration is particularly relevant because ovarian cancer progression is characterized by extensive dissemination within the peritoneal cavity. Śliwa et al. emphasized that successful ovarian cancer metastasis depends on considerable cellular plasticity, including transitions between epithelial and mesenchymal phenotypes and the ability of tumor cells to adopt different modes of migration during dissemination [18]. Moreover, intraperitoneal metastasis is strongly influenced by interactions between ovarian cancer cells and the peritoneal microenvironment, including resident macrophages and other immune components that may facilitate tumor implantation and metastatic progression [19]. The migratory phenotype of ovarian cancer cells is therefore regulated by complex interactions among tumor-intrinsic signaling pathways and microenvironmental factors affecting survival, cytoskeletal organization, adhesion, and motility.

In the present study, wound-healing responses showed distinct treatment-specific temporal patterns rather than uniform progressive inhibition. At 24 h, astaxanthin alone produced the most pronounced reduction in wound closure among the individual treatments, whereas carboplatin showed a more modest response. The everolimus–carboplatin combination also reduced wound closure, whereas the carboplatin–astaxanthin combination remained comparatively close to the untreated control. At 48 h, carboplatin and astaxanthin alone and the everolimus–carboplatin combination showed greater suppression of wound closure, whereas everolimus alone showed relatively limited inhibition. By 72 h, the response pattern had again changed: astaxanthin alone approached the untreated control, whereas the everolimus–carboplatin and carboplatin–astaxanthin combinations retained more pronounced inhibitory responses. These observations demonstrate that the individual agents and pairwise combinations differed not only in the magnitude but also in the temporal profile of their effects on wound closure. Because only one predefined working concentration of each agent was evaluated in this assay, these data cannot be used to infer dose-dependent effects on migration.

The biological basis for these divergent wound-healing profiles cannot be determined from the present experiments. Differences in the onset and persistence of responses to platinum-induced DNA damage, mTOR inhibition, and astaxanthin-associated cellular effects may contribute to the observed variation, but these explanations remain mechanistic hypotheses. Furthermore, scratch wound closure reflects both migration and proliferation. The treatment-associated differences in CCK-8 activity observed at the same experimental time points could therefore have contributed to differences in wound closure. For this reason, the present findings are more appropriately interpreted as treatment-specific effects on wound closure rather than definitive evidence of direct inhibition of migratory signaling. Confirmation using migration-specific methods, such as transwell migration or invasion assays, would be required to distinguish antimigratory activity from effects related to changes in cell number or proliferation.

Another important finding of the present study was the significant modulation of TNF-α and IL-1β following treatment. Chronic inflammation is increasingly recognized as an integral component of carcinogenesis and tumor progression. Zhao et al. [20] highlighted that persistent inflammatory signaling can promote tumor cell proliferation, survival, angiogenesis, invasion, and metastasis through interconnected pathways, including NF-κB, JAK/STAT, and PI3K/AKT signaling. This relationship appears particularly relevant in ovarian cancer, where the inflammatory tumor microenvironment contributes not only to disease progression but also to immune evasion and therapeutic resistance. Liu et al. [21] recently emphasized that inflammatory cytokines and their downstream signaling networks shape the ovarian cancer immune microenvironment and facilitate mechanisms of immune escape, suggesting that targeting inflammation-related pathways may represent a complementary therapeutic strategy.

Among these mediators, TNF-α and IL-1β are of particular interest because of their pleiotropic effects on both tumor cells and the surrounding microenvironment. Ovarian cancer cells can produce and respond to TNF-α, allowing this cytokine to participate in autocrine and paracrine signaling that may support tumor growth and amplify the production of other inflammatory mediators [8]. IL-1β similarly contributes to inflammatory signaling and may promote a microenvironment favorable to tumor progression. The potential relevance of these cytokines to ovarian cancer susceptibility is also supported by genetic evidence. Ahmed et al. [22] investigated polymorphisms in IL-1RN, IL-1β, and TNF-α and reported associations between selected inflammatory gene variants and ovarian cancer risk, further suggesting that alterations in IL-1β- and TNF-α-related pathways may be involved in ovarian carcinogenesis [23].

In our experiments, however, TNF-α and IL-1β responses varied according to treatment regimen and exposure duration rather than showing a uniform anti-inflammatory pattern. These changes should therefore not be interpreted as evidence that the tested agents consistently suppress inflammatory signaling. Instead, they indicate that exposure to the individual agents and their pairwise combinations was accompanied by alterations in inflammatory mediator levels. Although these changes occurred in parallel with alterations in CCK-8 activity and wound closure, the present data do not establish that TNF-α or IL-1β mediated either the viability or wound-healing responses.

The treatment-associated alterations in TNF-α and IL-1β observed in our study may therefore represent an additional biological dimension of the effects of carboplatin, everolimus, and astaxanthin. This interpretation is particularly interesting for astaxanthin because its anticancer activity has been linked to modulation of NF-κB signaling [7], whereas TNF-α is an important upstream and downstream component of NF-κB-associated inflammatory networks in ovarian cancer. In ovarian cancer cells, TNF-α has also been shown to regulate CXCR4 expression, a chemokine receptor associated with survival and metastatic potential [24]. More recent experimental evidence demonstrated that lysophosphatidic acid, an abundant mediator in ovarian cancer ascites, can induce TNF-α and IL-1β expression through inflammatory signaling involving NF-κB [9]. Accordingly, the concurrent changes in cytokines and functional cellular outcomes observed in the present study provide a rationale for future mechanistic investigation but cannot establish a causal pathway linking cytokine modulation to altered viability or wound closure.

The treatment-associated alterations in DEFA1, DEFB1, and DEFB2 observed in the present study may reflect changes in innate immune-related signaling accompanying cellular responses to treatment. Defensins have context-dependent roles in cancer and have been implicated in cellular proliferation, apoptosis, immune-cell recruitment, angiogenesis, and tumor progression [10,11,12]. Recent pan-cancer evidence has further demonstrated tumor-specific alterations in DEFB1 expression and associations with tumor biology and clinical outcomes [13]. In the present study, defensin levels changed in a treatment- and time-dependent manner, but their direction and magnitude were not uniform across treatment conditions. These observations suggest that defensin regulation may accompany the cellular response to pharmacological exposure; however, they do not demonstrate that defensins are responsible for the observed changes in viability or wound closure. Further studies integrating defensin gene expression, protein regulation, and downstream signaling are required to determine whether these peptides have a functional role or merely represent secondary treatment-associated responses.

### 3.1. Interpretation and Future Directions

Taken together, the present data should be viewed as a comparative characterization of individual and pairwise treatment responses rather than as evidence supporting a specific therapeutic combination. Carboplatin–everolimus represents concurrent platinum exposure and mTOR inhibition, whereas carboplatin–astaxanthin and everolimus–astaxanthin provide exploratory models for evaluating astaxanthin alongside conventional or targeted agents. Importantly, the magnitude and temporal pattern of CCK-8 activity, wound closure, and biomarker responses differed among these treatment strategies, and combination treatment was not uniformly more effective than the corresponding individual agents. Therefore, no pairwise regimen can be considered superior on the basis of the present experiments.

Future investigations should first establish broader concentration–response relationships and time-specific IC_50_ values for each agent. Formal drug interaction approaches should then be applied to determine whether individual combinations are synergistic, additive, or antagonistic. Additional ovarian cancer cell models with different molecular backgrounds would be important for assessing reproducibility and generalizability. Migration-specific assays, together with analyses of proliferation, apoptosis, cell cycle regulation, mTOR/NF-κB signaling, and relevant inflammatory pathways, would also help clarify the mechanisms underlying the observed temporal responses. Ultimately, in vivo validation would be necessary before considering any potential translational significance.

### 3.2. Limitations and Strengths of Study

Several limitations should be considered when interpreting the present findings. First, the experiments were conducted using a single ovarian cancer cell line, SKOV3, which does not fully represent the molecular and histological heterogeneity of ovarian cancer. Second, the study was limited to an in vitro model; therefore, the observed effects cannot be directly extrapolated to the complex tumor microenvironment or clinical setting. Third, although the pairwise combination treatments produced marked effects on cell viability and migration, formal drug interaction analyses were not performed; consequently, the findings should not be interpreted as evidence of pharmacological synergy or as demonstrating superiority of one combination over another. In addition, the concentration ranges evaluated in the viability assays were relatively limited and did not consistently bracket 50% cell viability; therefore, reliable IC_50_ values could not be determined. Future studies employing broader concentration ranges and nonlinear dose–response analyses are needed to establish time-specific IC_50_ values and provide a more comprehensive pharmacological characterization of these agents. Furthermore, the wound-healing assay may reflect both cellular migration and proliferation, and confirmation using migration-specific methods would strengthen these findings. Finally, although treatment-related changes in DEFA1, DEFB1, DEFB2, TNF-α, and IL-1β were demonstrated at the protein level, the underlying molecular pathways were not investigated.

Despite these limitations, the study has several strengths. The systematic evaluation of carboplatin, everolimus, and astaxanthin individually and in all three pairwise combinations allowed direct comparison of agents with distinct but potentially complementary mechanisms within the same experimental model. Moreover, assessment of both cell viability and migration provided functional information on different aspects of ovarian cancer cell behavior. The inclusion of defensins and pro-inflammatory cytokines further broadened the biological scope of the study by integrating innate immune- and inflammation-related responses with conventional cellular outcomes. The evaluation of multiple time points also enabled characterization of the temporal patterns of treatment effects. Together, this integrated experimental approach provides a basis for future mechanistic studies investigating the biological interactions within each pairwise treatment strategy and for subsequent validation in additional in vitro and in vivo ovarian cancer models.

### 3.3. Conclusions

In this in vitro SKOV3 model, carboplatin, everolimus, and astaxanthin, administered individually or in pairwise combinations, produced treatment- and time-dependent changes in CCK-8 activity, wound closure, and DEFA1, DEFB1, DEFB2, TNF-α, and IL-1β levels. The responses differed among individual agents and pairwise combinations and were not uniformly greater with combination treatment. CCK-8 activity does not directly measure proliferation, the wound-healing assay may have been influenced by both migration and proliferation, and formal drug interaction analyses were not performed; therefore, the present findings should be interpreted as preliminary comparative in vitro observations rather than evidence of synergistic or mechanistically defined antitumor effects. Further studies using broader dose ranges, additional ovarian cancer cell models, migration-specific and mechanistic assays, formal drug interaction analyses, and in vivo validation are required to determine the biological significance of these findings.

## 4. Materials and Methods

### 4.1. SKOV3 Cell Culture

The human ovarian cancer cell line SKOV3 (ATCC, Manassas, VA, USA) was cultured in RPMI-1640 medium (Gibco, Thermo Fisher Scientific, Waltham, MA, USA) supplemented with 10% fetal bovine serum (FBS; Sigma-Aldrich, St. Louis, MO, USA) and 1% penicillin–streptomycin (Sigma-Aldrich, St. Louis, MO, USA). Cells were maintained at 37 °C in a humidified incubator containing 5% CO_2_ and were passaged twice weekly to preserve exponential growth. For all experiments, cells were harvested at approximately 80% confluence.

Everolimus and astaxanthin stock solutions were prepared as concentrated stocks in dimethyl sulfoxide (DMSO) and diluted in complete culture medium immediately before use to obtain the desired working concentrations. Carboplatin stock solutions were prepared directly in complete culture medium and further diluted, when necessary, to the appropriate final concentrations immediately prior to treatment. For all functional assays, including cell viability and migration, cells were treated with the respective compounds for 24–72 h, depending on the experimental protocol. The final concentration of DMSO was maintained at 0.1% (*v*/*v*) in treatment groups containing everolimus and/or astaxanthin. A vehicle control containing 0.1% (*v*/*v*) DMSO was included to account for any solvent-related effects, whereas untreated control and carboplatin-treated cells were cultured in complete medium without DMSO. All experiments were performed independently in triplicate to ensure the reproducibility of the results.

SKOV3 cells were selected as the experimental model because they are widely used and well characterized in preclinical ovarian cancer research. Their high proliferative and migratory capacity provides a suitable in vitro system for evaluating changes in tumor cell growth and migration following pharmacological interventions. In addition, the p53-null background and relative resistance of SKOV3 cells to apoptosis make this model particularly relevant for investigating drug responses and the effects of combination treatment strategies. These characteristics allowed us to assess the effects of everolimus, carboplatin, and astaxanthin on different aspects of ovarian cancer cell behavior. Nevertheless, ovarian cancer is a biologically heterogeneous disease characterized by considerable molecular and histopathological diversity. Thus, findings obtained from a single cell line cannot fully represent the complex biology of ovarian cancer. Future studies using additional ovarian cancer cell lines with different genetic and molecular profiles, together with in vivo models, are warranted to validate the present findings and determine their broader biological and translational relevance.

### 4.2. Cell Viability Assays

SKOV3 cells were seeded into 96-well plates at a density of 1.5 × 10^4^ cells per well and allowed to adhere for 24 h under standard culture conditions. Based on preliminary experiments and the published literature, cells were subsequently treated with increasing concentrations of everolimus (10–100 nM and 1 μM), carboplatin (10–200 μM), or astaxanthin (10–100 μM) for up to 72 h. The selected concentration ranges were intended to characterize concentration- and time-dependent viability responses and to guide the selection of concentrations for subsequent pairwise combination experiments. In the combination viability experiments, 75 μM carboplatin was additionally evaluated as an intermediate exploratory concentration. For the subsequent wound-healing and biomarker assays, standardized working concentrations of 100 μM carboplatin, 100 μM astaxanthin, and 100 nM everolimus were used consistently for the individual treatments and their pairwise combinations across all time points. The selection of 100 μM carboplatin for these downstream assays was intended to provide a uniform experimental condition and should not be interpreted as indicating that 75 μM carboplatin was ineffective or inferior. These concentration ranges were not designed for formal IC_50_ determination. Because the tested concentrations did not consistently encompass viability values above and below 50% for all compounds and time points, IC_50_ values were not calculated. Cell viability was evaluated using the Cell Counting Kit-8 (CCK-8; Abbkine, Wuhan, China) according to the manufacturer’s instructions. Briefly, 10 μL of CCK-8 reagent was added to each well, followed by incubation for 3 h at 37 °C. The absorbance was measured at 450 nm using a microplate reader (BioTek Instruments, Winooski, VT, USA). Cell viability was calculated relative to the untreated control group, which was defined as having 100% viability. All experiments were performed independently in triplicate, and the results are presented as the mean ± standard deviation (SD).

### 4.3. Wound Healing Assay

SKOV3 cells were seeded into 6-well plates and cultured until approximately 80% confluence was reached. A standardized linear scratch was generated in the cell monolayer using a sterile 100 μL pipette tip held at a constant perpendicular angle to ensure a uniform initial wound width across all wells. Detached cells and cellular debris were removed by washing the wells twice with 1× phosphate-buffered saline (PBS). Subsequently, cells were treated with the indicated concentrations of everolimus, carboplatin, or astaxanthin and incubated for the designated experimental period.

Images of the wounded areas were captured at 0 h and at the indicated time points using an inverted microscope. Cell migration was quantified by measuring the wound gap width (μm) using the MShot Image Analysis System software V1.1.6_A (MShot.Main, Micro-Image Analysis System). For each experimental group, three representative microscopic fields were analyzed, and the mean value was used for subsequent analysis. Wound closure was determined by measuring the average distance between the wound edges at multiple points and normalizing the values to the initial wound width at 0 h.

The percentage of migration inhibition was calculated relative to the untreated control group using the following formula:

Migration inhibition (%) = [1 − (wound closure of the treated group/wound closure of the control group)] × 100

### 4.4. Assay of Biochemical Parameters

The protein levels of human α-defensin 1 (DEFA1), β-defensin 1 (DEFB1), β-defensin 2 (DEFB2), tumor necrosis factor-alpha (TNF-α), and interleukin-1 beta (IL-1β) were quantified in SKOV3 cell lysates using commercially available sandwich enzyme-linked immunosorbent assay (ELISA) kits (BT Laboratory, Jiaxing, China), according to the manufacturer’s instructions. The catalog numbers were E4726Hu for DEFA1, E6426Hu for DEFB1, E6427Hu for DEFB2, E0082Hu for TNF-α, and E0143Hu for IL-1β. Cells were lysed by repeated freeze–thaw cycles in PBS and centrifuged, and the resulting supernatants were used for analysis. Samples were incubated with the corresponding detection antibodies and streptavidin-HRP for 60 min at 37 °C, followed by washing and substrate incubation. Absorbance was measured at 450 nm, and analyte concentrations were calculated from standard curves.

### 4.5. Statistical Analysis

Statistical analyses were performed using GraphPad Prism version 8.0.1 (GraphPad Software, San Diego, CA, USA). Normality of the data distribution was evaluated using the Kolmogorov–Smirnov test prior to statistical analysis. Since the data did not meet the assumptions of normality, non-parametric tests were applied. Comparisons among multiple independent groups were performed using the Kruskal–Wallis test. When a statistically significant difference was detected, Dunn’s multiple comparisons post hoc test was used to identify differences between individual groups. Results were expressed as mean ± standard deviations where appropriate. A *p*-value of <0.05 was considered statistically significant.

## Data Availability

The datasets generated and/or analyzed in the current study are available from the corresponding author upon reasonable request. All data supporting the findings of this study are included within the article.
